# Search for Therapeutic Agents for Cardiac Arrest Using a Drug Discovery Tool and Large-Scale Medical Information Database

**DOI:** 10.3389/fphar.2019.01257

**Published:** 2019-11-08

**Authors:** Yoshito Zamami, Takahiro Niimura, Toshihiro Koyama, Yuta Shigemi, Yuki Izawa-Ishizawa, Mizuki Morita, Ayako Ohshima, Keisaku Harada, Toru Imai, Hiromi Hagiwara, Naoto Okada, Mitsuhiro Goda, Kenshi Takechi, Masayuki Chuma, Yutaka Kondo, Koichiro Tsuchiya, Shiro Hinotsu, Mitsunobu R. Kano, Keisuke Ishizawa

**Affiliations:** ^1^Department of Clinical Pharmacology and Therapeutics, Institute of Biomedical Sciences, Tokushima University Graduate School, Tokushima, Japan; ^2^Department of Pharmacy, Tokushima University Hospital, Tokushima, Japan; ^3^Department of Clinical Evaluation and Development of Pharmaceutical Biomedicine, Graduate School of Medicine, Dentistry, and Pharmaceutical Sciences, Okayama University, Kita-ku, Japan; ^4^Department of Emergency Pharmaceutical Science, Graduate School of Medicine, Dentistry and Pharmaceutical Sciences, Okayama University, Okayama, Japan; ^5^AWA Support Center, Tokushima University, Tokushima, Japan; ^6^Biomedical Informatics, Graduate School of Interdisciplinary Science and Engineering in Health Systems, Okayama University, Kita-ku, Japan; ^7^Department of Pharmaceutical Biomedicine, Graduate School of Medicine, Dentistry, and Pharmaceutical Sciences, Okayama University, Kita-ku, Japan; ^8^Department of Pharmacy, Kitakyushu City Yahata Hospital, Kitakyushu-shi, Japan; ^9^Department of Pharmacy, Nihon University Itabashi Hospital, Tokyo, Japan; ^10^Department of Medical Innovation, Nagoya City University Graduate School of Medical Sciences, Nagoya, Japan; ^11^Clinical Trial Center for Developmental Therapeutics, Tokushima University Hospital, Tokushima, Japan; ^12^Department of Emergency and Critical Care Medicine, Juntendo University Urayasu Hospital, Urayasu, Japan; ^13^Department of Medical Pharmacology, Tokushima University Graduate School of Biomedical Sciences, Tokushima, Japan; ^14^Department of Biostatistics, Sapporo Medical University, Sapporo, Japan

**Keywords:** cardiac arrest, drug repositioning, claims database, drug discovery tool, vasodilator

## Abstract

The survival rate of cardiac arrest patients is less than 10%; therefore, development of a therapeutic strategy that improves their prognosis is necessary. Herein, we searched data collected from medical facilities throughout Japan for drugs that improve the survival rate of cardiac arrest patients. Candidate drugs, which could improve the prognosis of cardiac arrest patients, were extracted using “TargetMine,” a drug discovery tool. We investigated whether the candidate drugs were among the drugs administered within 1 month after cardiac arrest in data of cardiac arrest cases obtained from the Japan Medical Data Center. Logistic regression analysis was performed, with the explanatory variables being the presence or absence of the administration of those candidate drugs that were administered to ≥10 patients and the objective variable being the “survival discharge.” Adjusted odds ratios for survival discharge were calculated using propensity scores for drugs that significantly improved the proportion of survival discharge; the influence of covariates, such as patient background, medical history, and treatment factors, was excluded by the inverse probability-of-treatment weighted method. Using the search strategy, we extracted 165 drugs with vasodilator activity as candidate drugs. Drugs not approved in Japan, oral medicines, and external medicines were excluded. Then, we investigated whether the candidate drugs were administered to the 2,227 cardiac arrest patients included in this study. The results of the logistic regression analysis showed that three (isosorbide dinitrate, nitroglycerin, and nicardipine) of seven drugs that were administered to ≥10 patients showed significant association with improvement in the proportion of survival discharge. Further analyses using propensity scores revealed that the adjusted odds ratios for survival discharge for patients administered isosorbide dinitrate, nitroglycerin, and nicardipine were 3.35, 5.44, and 4.58, respectively. Thus, it can be suggested that isosorbide dinitrate, nitroglycerin, and nicardipine could be novel therapeutic agents for improving the prognosis of cardiac arrest patients.

## Introduction

Despite advances in treatment, cardiac arrest still results in a high mortality rate. In the United States alone, more than 550,000 cardiac arrest cases are reported annually, with the survival discharge rate being only 12% and 24.8% for out-of-hospital and in-hospital patients, respectively (The American Heart Association, 2013). Considering the increase in aging population, the number of cardiac arrest patients is also expected to increase; hence, there is an urgent need to develop a treatment that improves the prognosis of patients suffering cardiac arrest.

Cardiac arrest damages heart functions and those of other organs. Particularly, it results in myocardial dysfunction at the early stages after resuscitation; moreover, circulation becomes very unstable (Jentzer et al., 2015). Myocardial dysfunction is associated with early death, and if improvement in cardiac function cannot be achieved within 24 h, conditions such as multiple organ failure, which have poor prognosis, are known to occur (Laurent et al., 2002). Therefore, normalization of hemodynamics is very important for improving the prognosis after cardiac arrest.

In recent years, drug repositioning (DR) has attracted attention as a strategy of drug development. In the DR approach, new pharmacological effects of already approved drugs with known safety for humans are identified. These drugs are then used as new therapeutic agents for other diseases according to the identified effect (Ashburn and Thor, 2004; Zamami et al., 2017). The number of new drugs introduced in the market is decreasing yearly because the development of new drugs is expensive and time-consuming (Reuters, 2011). The main advantage of using the DR approach is that it can reduce drug development time and cost. Moreover, it reduces the risk of development failure due to unintended adverse effects and pharmacokinetic problems in the clinical trial stage. Various types of big data are now available, and utilization of these data can be highly useful for the DR approach. We anticipate that DR can contribute to the development of therapeutic agents that improve prognosis of cardiac arrest patients.

One such big data in the field of life science is the drug discovery science database. It includes a wide variety of information such as chemical structures, physical properties, pharmacological actions, and genes related to diseases. TargetMine has been developed by the National Institutes of Biomedical Innovation, Health and Nutrition (Japan). It integrates more than 30 databases on bioinformatics worldwide (Chen et al., 2016). It can be used to identify related diseases and drugs based on a specific gene. Similarly, a representative medical information big data for the medical sciences is Japan Medical Data Center (JMDC) Claims Database. It is a database of 5.6 million cases collected from Japan’s health insurance association, which includes information on the diagnosis of diseases and prescription medicines and reflects the actual clinical practices. Using these databases, it is possible to evaluate the efficacy and safety of drugs in the real-world setting (Sugiyama et al., 2016; Yokoyama et al., 2018).

It is difficult to develop an experimental drug for cardiac arrest due to the urgency of treatment and ethical problems. However, we can use the DR approach by utilizing drug discovery science databases and medical information databases to identify candidate drugs expected to be effective in humans. Therefore, in this study, we aimed to search for drugs that can improve the survival rate of cardiac arrest patients using drug discovery tools and large-scale medical databases through the DR approach.

## Methods

### Extraction of Candidate Drugs by Targetmine

TargetMine internally integrates and combines various data from around the world. It is possible to use it for drug development by simultaneously specifying conditions such as target genes, proteins, and pharmacological actions, and thus simplifying complicated tasks. Compared with other data warehousing tools, TargetMine has simpler operation, and users can collect and prioritize information quickly and efficiently without the need for special programming skills (Chen et al., 2011; Chen et al., 2016). The occurrence of cardiac arrest causes systemic circulatory failure and hypoxia. Therefore, we focused our literature search on vasodilators because of their use in maintaining circulation after resumption of heart functioning (Yuuki et al., 1991). In this study, we used Anatomical Therapeutic Chemical Classification System (ATC) codes (widely used as a method to classify medicines according to efficacy, site of action, target organ, and chemical characteristics) as an extraction method of candidate drugs. We searched for pharmaceutical ingredients with an ATC code associated with vasodilator effect (**Supplementary Table 1**). TargetMine is a comprehensive tool and is suitable for this study because it can be used to extract information on drugs with pharmacological actions that correspond to specific ATC classifications.

### Analysis of Large-Scale Medical Information

The claims database used in analysis was provided by the Japan Medical Data Center (JMDC), which includes approximately 3 million cases (as of November 2015) of receipt information. Information from medical hospital receipts, Diagnosis Procedure Combination (DPC) receipts, medical out-of-hospital receipts, and dispensing receipts was integrated. It was possible to obtain information, including disease name, medical treatment, and the dispensed items, from the insurance documents (**Supplementary Table 2–8**). Moreover, since a unique ID is given to each participant, if one patient visits multiple medical institutions, it is possible to trace a series of processes from the occurrence of a disease to its convergence. Nevertheless, since all data are highly encrypted unlinkable anonymized information, it is not possible to identify an individual. The receipt dataset used in this study is provided in seven files: “patient information,” “receipt information,” “facility information,” “doctor information,” “injury information,” “pharmaceutical information,” and “medical treatment information.” The items contained in each file and their contents are shown in **Supplementary Tables 2, 3, 4, 5, 6, 7, and 8**. It should be noted that the diagnostic names in this dataset are used by the WHO for referring to causes of death and disease. The classification was based on the 10th edition of “International Statistical Classification of Diseases and Related Health Problems” (ICD-10). Drugs were classified according to the Anatomical Therapeutic Chemical Classification System (ATC classification) developed by the European Pharmaceutical Market Research Association (EphMRA). From January 2005 to May 2014, patients with the following (ICD-10) codes were classified as cardiac arrest patients (*n* = 2,546): I469 for cardiac arrest, I472 for pulseless ventricular tachycardia, I490 for transient ventricular fibrillation; and Japan specific medical action codes: J046 for non-thoracotomy heart massage, J047 for counter shock. Among these patients, those with traumatic cardiac arrest or <18 years of age or unconfirmed diagnosis or having missing data were excluded. Finally, 2,227 patients were included in the study. Cardiac arrest date was defined as the date at which the diagnosis of cardiac arrest was started; if the diagnosis date was unknown, the date on which the practice for cardiac arrest was performed was used. If multiple cardiac arrests occurred during the study period, the first date was taken as the patient’s cardiac arrest date. We used the presence or absence of “the fee for providing treatment information at discharge” in claim data to define survival discharge. Patients who were charged this fee within 1 year from the day of cardiac arrest were defined as survival discharge patients. The data were processed using Microsoft Access 2013, and the information contained in the seven files described above was linked based on the patient ID. Because data about “birth date,” “join date,” “JMDC data start date,” and “medical treatment date” could not be obtained, these dates were all set to the first day of that month. In addition, “the withdrawal date” and “JMDC data end date” were set to the last day of that month. In case the “medical care start date,” the “prescription date of medicine,” or the “treatment date” were blank, the “consultation date” was used instead.

### Narrowing Down Candidate Drugs

**Figure 1:**
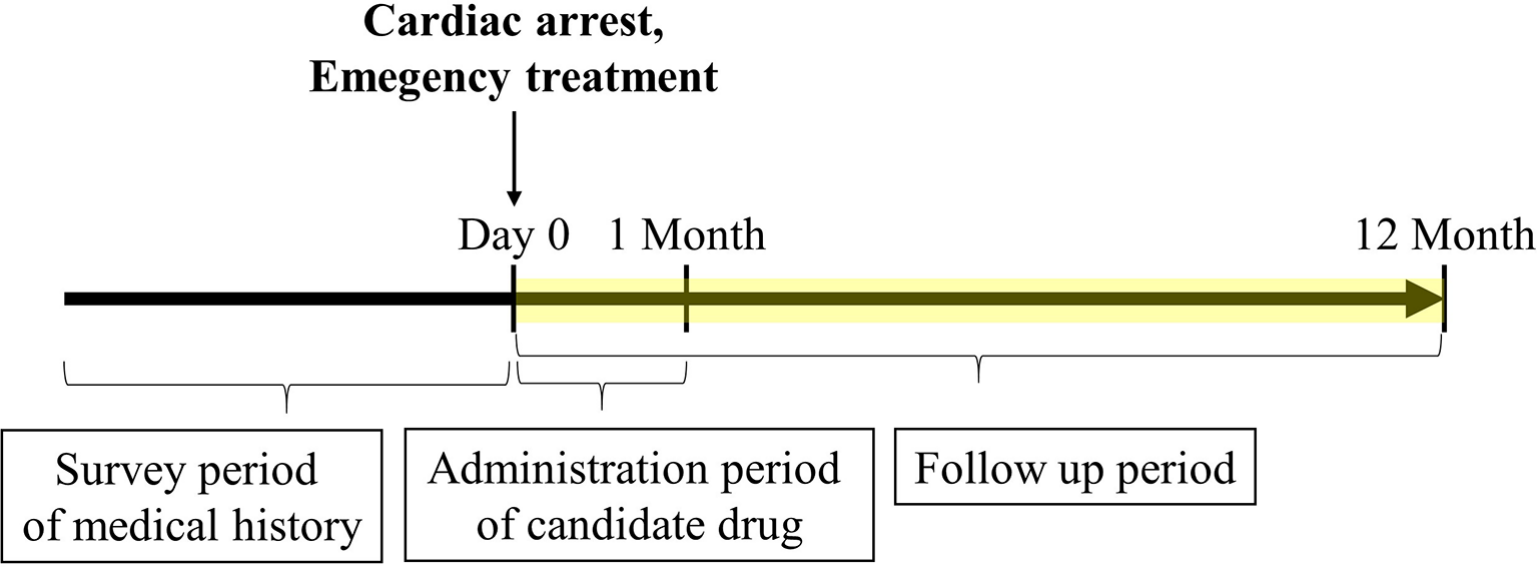
Timeline of claims data analysis. The cardiac arrest date was set as day 0. The frequency of survival discharge within 1 year after cardiac arrest was determined. Only administration of candidate drugs limited to 1 month or less after cardiac arrest was included. The medical history and emergency treatment recorded before cardiac arrest and on the day of cardiac arrest were included, respectively.

First, the drugs that were not currently approved in Japan were excluded. Parenteral administration suitable for the the patients with cardiac arrest because of they suffer from sever condition and are unconscious. Therefore, the drugs for which no injectable form was available were excluded. Subsequently, information of drugs administered within 1 month after cardiac arrest was extracted from 2,227 cardiac arrest cases identified as target patients. Of these, drugs administered in ≤10 patients were excluded as statistically reliable results could not be obtained when the number of patients was very small. The correlation between the administration of the candidate drugs and the survival discharge within 1 year was analyzed by logistic regression analysis. The administration of each candidate drug (diltiazem, disopyramide, flecainide, verapamil, isosorbide nitrate, nicardipine, and nitroglycerin) within 1 month from the cardiac arrest date was set as the explanatory variable, and the survival discharge within 1 year from the cardiac arrest date was set as the objective variable (**Figure 1**). The adjusted odds ratios (ORs) were calculated for the drugs showing a significant relationship with survival discharge in simple logistic regression analysis. The covariates used for the adjustment are shown n **Supplementary Table 9**.

### Statistical Analysis

Continuous variables are presented as the mean ± standard deviation (SD) and categorical variables by using frequencies and percentages. To compare the effects of administration of each drug on survival discharge, simple logistic regression was performed with administration of diltiazem, disopyramide, flecainide, verapamil, isosorbide dinitrate, nicardipine, and nitroglycerin as covariates (**Table 3**). Isosorbide dinitrate, nitroglycerin, and nicardipine were divided into two groups based on the presence or absence of administration. Standardized mean differences (**Tables 4**, **5**, **6**) were calculated after adjusting each covariate using the inverse probability of treatment weighting (IPTW) method, and the variation of the covariates in both groups was examined. Using the IPTW method, adjusted ORs were calculated for comparing the rates of survival discharge between the two groups (**Table 7**). Analyses were performed using R statistical software version 3.1.2., and statistical significance was defined as a *p* < 0.05.

### Ethics Statement

This study was carried out in accordance with the recommendations of the Ethical Guidelines for Epidemiological Research by the Ministry of Health, Labour and Welfare. The protocol was approved by the ethics committees of Okayama University Graduate School of Medicine, Dentistry and Pharmaceutical Sciences and Okayama University Hospital (no. 105056). One cannot identify individuals from JMDC’s claims database because all personal data including names are deleted and converted to an ID that cannot be linked with any personal information. Since this study was an observational study with anonymized information, no treatment intervention, and no collection of human samples, the requirement of obtaining informed consent was waived by the ethics committee.

## Results

### Candidate Drug Data Extraction Using Targetmine

Of the drugs shortlisted using TargetMine, drugs with ATC code “C01B” included 51 drugs, “C01DA,” “C02CA,” and “C02CC” included 10 drugs each, “C04” included 52 drugs, and “C08” included 44 drugs. The total number of drugs excluding duplicates was 165 (**Table 1**).

**Table 1:** Names of shortlisted candidate drugs extracted using TargetMine (excluding duplicates).

Drug names (165)			
Ajmaline	Doxazosin	Lidocaine hydrochloride	Propafenone
Amiodarone	Doxazosin mesylate	Lidocaine hydrochloride monohydrate	Propafenone hydrochloride
Amiodarone hydrochloride	Dronedarone	Lidoflazine	Propyl nitrate
Amlodipine	Dronedarone hydrochloride	Lorajmine hydrochloride	Quinidine
Amlodipine besylate	Encainide	Lorcainide	Quinidine gluconate
Amlodipine maleate	Encainide hydrochloride	Lorcainide hydrochloride	Quinidine phenylethyl barbiturate
Aprindine	Ergoloid mesylates	Manidipine	Quinidine polygalacturonate
Aprindine hydrochloride	Erythrityl tetranitrate	Manidipine hydrochloride	Quinidine sulfate
Azapetine	Ethacizine	Methyl nicotinate	Sparteine
Bamethan	Fasudil	Mexiletine	Sparteine sulfate
Bamethan sulfate	Fasudil hydrochloride	Mexiletine hydrochloride	Suloctidil
Barnidipine	Fasudil hydrochloride hydrate	Mibefradil	Tedisamil
Barnidipine hydrochloride	Felodipine	Mibefradil dihydrochloride	Tedisamil sesquifumarate
Bencyclane	Fendiline	Moricizine	Tocainide
Bencyclane fumarate	Fendiline hydrochloride	Moricizine hydrochloride	Tocainide hydrochloride
Benidipine	Flecainide	Moxisylyte	Tolazoline
Benidipine hydrochloride	Flecainide acetate	Moxisylyte hydrochloride	Tolazoline hydrochloride
Benzyl nicotinate	Gallopamil	Nafronyl oxalate	Trimazosin
Bepridil	Gallopamil hydrochloride	Naftidrofuryl	Trimazosin hydrochloride
Bepridil hydrochloride	Guanazodine	Niacin	Trolnitrate phosphate
Bethanidine sulfate	Guanethidine	Nicardipine	Urapidil
Bretylium tosylate	Guanethidine sulfate	Nicardipine hydrochloride	Urapidil hydrochloride
Buflomedil	Guanoclor sulfate	Nicergoline	Verapamil
Buflomedil hydrochloride	Guanoxabenz	Nicergoline tartrate	Verapamil hydrochloride
Bunaftine	Guanoxan sulfate	Nicotinyl alcohol	Vernakalant hydrochloride
Buphenine	Hydroquinidine	Nifedipine	Vinburnine
Butalamine	Hydroquinidine hydrochloride	Nilvadipine	Vincamine
Cetiedil citrate	Ibutilide	Nimodipine	Vincamine hydrochloride
Ciclonicate	Ibutilide fumarate	Nisoldipine	Visnadine
Cifenline	Ifenprodil	Nitrendipine	Xanthinol niacinate
Cifenline succinate	Ifenprodil tartrate	Nitroglycerin	
Cilnidipine	Indoramin	Nylidrin hydrochloride	
Cinepazide maleate	Indoramin hydrochloride	Pentaerythritol tetranitrate	
Clevidipine	Inositol niacinate	Pentifylline	
Cyclandelate	Isosorbide dinitrate	Pentoxifylline	
Debrisoquin	Isosorbide mononitrate	Perhexiline	
Debrisoquin sulfate	Isoxsuprine	Perhexiline maleate	
Dihydroergocristine	Isoxsuprine hydrochloride	Phenoxybenzamine	
Dihydroergocristine mesilate	Isoxsuprine lactate	Phenoxybenzamine hydrochloride	
Diltiazem	Isradipine	Phentolamine	
Diltiazem hydrochloride	Kallidinogenase	Phentolamine mesylate	
Diltiazem maleate	Lacidipine	Prazosin	
Disopyramide	Lercanidipine	Prazosin hydrochloride	
Disopyramide phosphate	Lercanidipine hydrochloride	Procainamide	
Dofetilide	Lidocaine	Procainamide hydrochloride	

**Figure 2:**
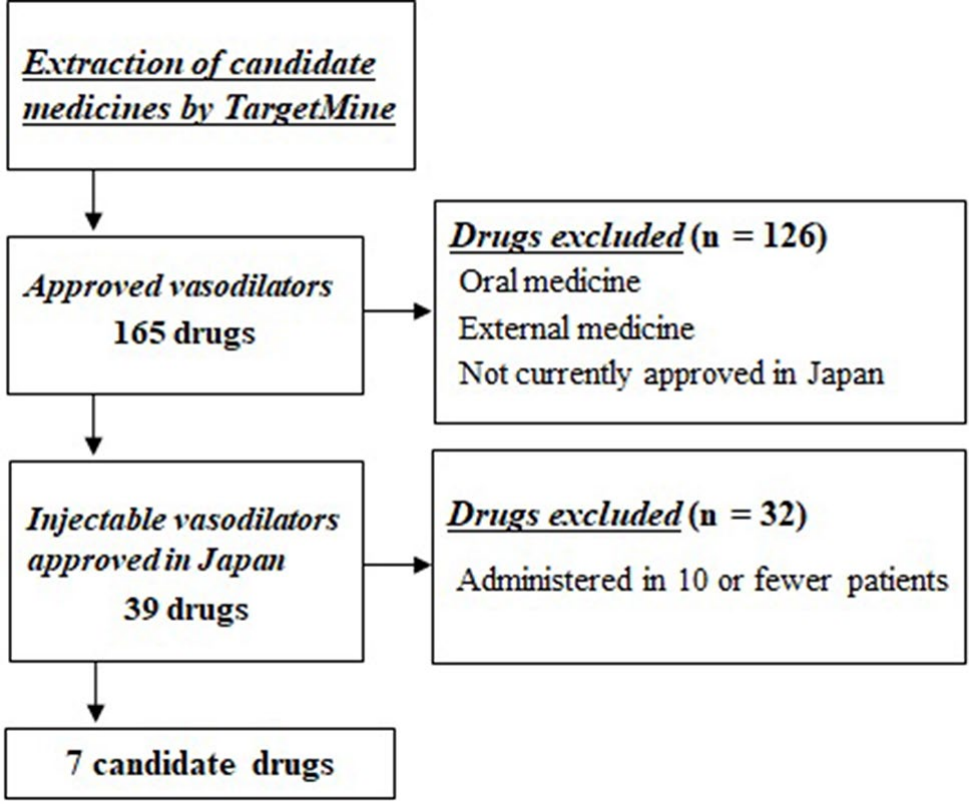
Selection flowchart of candidate drug. First, 165 approved drugs having vasodilator activity were extracted using the drug discovery tool TargetMine. Next, drugs that were not approved in Japan, oral drugs, and external drugs were excluded, and seven drugs were selected that were administered to ≥10 patients within 1 month of cardiac arrest.

Of these 165 candidate drugs, 39 were excluded, including those not either approved in Japan or those for which no injectable form was available. After further shortlisting for the drugs administered within 1 month from the day of cardiac arrest, 11 drugs were obtained. Finally, after exclusion of drugs that were used by ≤10 patients and those included in statistical analysis as covariates, seven drugs were shortlisted as the drug candidates for this study. The names and frequencies of drug administration of each drug are shown in **Table 2**, and the process of data extraction for drugs is shown in **Figure 2**.

**Table 2:** Number of cardiac arrest patients administered each drug.

Drug name	Number of patients administered	Drug name	Number of patients administered	Drug name	Number of patients administered
Ajimarin	0	Diltiazem	45	Fassille	5
Apringin	9	Cilnidipine	0	Felodipine	0
Amiodarone	119	Doxazosin	0	Phentolamine	0
Amlodipine	0	Trazoline	0	Prazosin	0
Isoxsuprine	0	Niacin	0	Flecainide	28
Inositol	0	Nicardipine	98	Procainamide	10
Ifenprodil	0	Nicergoline	0	Propafenone	0
Urapidil	0	Nisoldipine	0	Benidipine	0
Kallidinogenase	0	Nitrangepin	0	Bepridil	0
Quinidine	0	Nitroglycerin	103	Verapamil	134
Guanethidine	0	Nifedipine	0	Mexiletine	2
Disopyramide	32	Nilvadipine	0	Lidocaine	559
Dihydroergocristine	0	Barnidipine	0	Isosorbide nitrate	164

### Relationship Between Drug Use and Survival Discharge

Logistic regression analysis indicated significant positive correlation between the administration of candidate drugs (the explanatory variable) and survival discharge within 1 year (the objective variable) for isosorbide dinitrate (OR = 3.80, 95% CI = 2.52–5.66), nitroglycerin (OR = 2.79, 95% CI = 1.59–4.74), and nicardipine (OR = 2.27, 95% CI = 1.25-3.99) (**Table 3**).

**Table 3:** Relationship between each drug and survival discharge.

Explanatory variable	*p* value	Odds ratio (95% CI)
Diltiazem	0.573	0.76 (0.27–1.87)
Disopyramide	0.199	1.92 (0.64–4.82)
Flecainide	0.272	1.79 (0.57–4.74)
Verapamil	0.181	1.45 (0.82–2.43)
Isosorbide nitrate	<0.001	3.80 (2.52–5.66)
Nicardipine	0.006	2.27 (1.25–3.99)
Nitroglycerin	<0.001	2.79 (1.59–4.74)

Propensity score matching was performed to minimize patient background bias in the treatment and non-treatment groups. Standardized effects of each covariate before and after adjustment using the IPTW method are shown in **Tables 4**, **5**, **6**. The values of standardized effect decreased after adjustment compared with the values before adjustment, and the patient background bias was uniform. The adjusted odds ratios after adjustment with the IPTW method were as follows: isosorbide dinitrate, 3.35 (95% CI = 1.79–6.26); nitroglycerin, 5.44 (95% CI = 3.06–9.68); and nicardipine, 4.58 (95% CI = 2.53–8. 28) (**Table 7**). These findings suggest that the survival discharge rate was significantly high in all three treatment groups.

**Table 4:** Comparison of isosorbide dinitrate covariates.

Background factor	(number of patients) [SD]		Standardized effect values	
	Isosorbide nitrate administration group (*n* = 164)	Non-administration group (*n* = 2,063)	Before adjustment	After adjustment
Male	83.5% (137)	71.8% (1482)	0.26	0.07
Age	56.1 [10.5]	55.0 [13.3]	0.09	0.08
**Medical history**				
Ischemic heart disease	26.8% (44)	18.7% (385)	0.21	0.01
Cerebrovascular disease	9.1% (45)	12.2% (252)	0.09	0.02
Kidney disease	6.7% (11)	8.8% (181)	0.07	0.08
Liver disease	15.9% (26)	19.0% (392)	0.08	0.12
Chronic lung disease	22.0% (36)	24.6% (507)	0.06	0.1
Heart failure	19.5% (32)	24.5% (506)	0.12	0.11
Diabetes mellitus	34.1% (56)	23.8% (491)	0.24	0.04
High blood pressure	42.7% (70)	39.1% (806)	0.07	0.11
Hyperlipidemia	28.7% (47)	20.2% (416)	0.21	0.1
Malignant neoplasm	10.4% (17)	20.2% (417)	0.25	0.12
**Emergency treatment factor**				
Out-of-hospital cardiac arrest	25.6% (42)	18.1% (373)	0.19	0.13
Average number of instances of defibrillation	0.40 [0.49]	0.29 [0.49]	0.23	0.17
Tracheal intubation	27.4% (45)	20.5% (422)	0.17	0.04
Artificial respiration	32.9% (54)	27.5% (567)	0.12	0.06
Hypothermia	9.1% (15)	1.0% (21)	0.64	0.03
Average number of adrenaline doses	0.64 [1.78]	1.38 [3.55]	0.21	0.04
Vasopressin	0.61% (1)	0.5% (11)	0.01	0.05
Amiodarone	20.1% (33)	2.4% (49)	0.94	0.01
Lidocaine	17.7% (29)	2.9% (60)	0.75	0.01
Nifekalant	2.4% (4)	0.5% (10)	0.25	0.23

**Table 5:** Comparison of nitroglycerin covariates.

Background factor	(number of people) [SD]		Standardized effect values	
	Nitroglycerin administration group (*n* = 103)	Non-administration group (*n* = 2,124)	Before adjustment	After adjustment
Male	79.6% (82)	72.4% (1537)	0.16	0.01
Age	56.4 [12.6]	55.0 [13.2]	0.11	0.08
**Medical history**				
Ischemic heart disease	30.1% (31)	18.7% (398)	0.29	0.12
Cerebrovascular disease	14.6% (15)	11.9% (252)	0.08	0.09
Kidney disease	12.6% (13)	8.4% (179)	0.15	0.05
Liver disease	13.6% (14)	19.0% (404)	0.14	0.01
Chronic lung disease	24.3% (25)	24.4% (518)	0	0
Heart failure	24.3% (25)	24.2% (513)	0	0.06
Diabetes mellitus	26.2% (27)	24.5% (520)	0.04	0.01
High blood pressure	49.5% (51)	38.8% (825)	0.22	0.05
Hyperlipidemia	22.3% (23)	20.7% (440)	0.04	0
Malignant neoplasm	12.6% (13)	19.8% (421)	0.18	0.03
**Emergency treatment factor**				
Out-of-hospital cardiac arrest	23.3% (24)	18.4% (391)	0.13	0.05
Average number of instances of defibrillation	0.42 [0.85]	0.29 [0.46]	0.26	0.06
Tracheal intubation	27.2% (28)	20.7% (439)	0.16	0.03
Artificial respiration	41.7% (43)	27.2% (578)	0.32	0.04
Hypothermia	7.8% (8)	1.3% (28)	0.51	0.03
Average number of adrenaline doses	1.12 [2.88]	1.34 [3.48]	0.06	0.06
Vasopressin	0.0% (0)	0.6% (12)	0.08	0.08
Amiodarone	13.6% (14)	3.2% (68)	0.55	0.11
Lidocaine	27.2% (28)	2.9% (61)	1.24	0.08
Nifekalant	1.9% (2)	0.6% (12)	0.17	0.04

**Table 6:** Comparison of nicardipine covariates.

Background factor	(number of people) [SD]		Standardized effect values	
	Nicardipine administration group (*n* = 98)	Non-administration group (*n* = 2,129)	Before adjustment	After adjustment
Male	76.5% (75)	72.5% (1544)	0.09	0.05
Age	58.0 [10.9]	54.9 [13.2]	0.23	0
**Medical history**				
Ischemic heart disease	28.6% (28)	18.8% (401)	0.25	0.07
Cerebrovascular disease	18.4% (18)	11.7% (249)	0.21	0.03
Kidney disease	8.2% (8)	8.6% (184)	0.02	0.01
Liver disease	17.3% (17)	18.8% (401)	0.04	0
Chronic lung disease	24.5% (24)	24.4% (519)	0	0.05
Heart failure	26.5% (26)	24.0% (512)	0.06	0.09
Diabetes mellitus	28.6% (28)	24.4% (519)	0.1	0.15
High blood pressure	53.1% (52)	38.7% (824)	0.29	0.05
Hyperlipidemia	27.6% (27)	20.5% (436)	0.17	0.03
Malignant neoplasm	18.4% (18)	19.5% (416)	0.03	0.1
**Emergency treatment factor**				
Out-of-hospital cardiac arrest	28.6% (28)	18.2% (387)	0.27	0.06
Average number of instances of defibrillation	0.32 [0.49]	0.28 [0.49]	0.04	0.03
Tracheal intubation	35.7% (35)	20.3% (432)	0.38	0.04
Artificial respiration	53.1% (52)	26.7% (569)	0.59	0.11
Hypothermia	8.2% (8)	1.3% (28)	0.54	0.02
Average number of adrenaline doses	1.78 [3.72]	1.31 [3.44]	0.14	0.01
Vasopressin	0.0% (0)	0.6% (12)	0.08	0.08
Amiodarone	9.2% (9)	3.4% (73)	0.31	0.03
Lidocaine	27.6% (27)	2.9% (62)	1.26	0.01
Nifekalant	3.1% (3)	0.5% (11)	0.32	0.05

**Table 7:** Covariate adjusted odds ratios.

Drug name	Adjusted odds ratio (95% CI)	*p* value
Isosorbide nitrate	3.35 (1.79–6.26)	<0.001
Nitroglycerin	5.44 (3.06–9.68)	<0.001
Nicardipine	4.58 (2.53–8.28)	<0.001

## Discussion

Even after matching on covariates using propensity scores, the odds ratios for survival discharge within 1 year after cardiac arrest were significantly high in each of the isosorbide dinitrate, nitroglycerin, and nicardipine administration groups. This indicated that all three drugs improved the survival rate on hospital discharge.

To date, no large-scale clinical studies demonstrating the efficacy of nitroglycerin, isosorbide dinitrate, and nicardipine in cardiac arrest patients have been reported. However, some case reports were published reporting resuscitation being achieved by the administration of nitroglycerin in refractory cardiac arrest (Ward and Reid, 1984; Osada et al., 2000; Stefaniotou et al., 2014). Moreover, it was reported that acute coronary syndrome (ACS), which occurs after resuscitation from cardiac arrest, and cardiac arrest caused by ACS were improved by the administration of isosorbide dinitrate (Heming et al., 2010; Manzo-Silberman et al., 2010). In animal experiments using the heart of a paralyzed rat, nicardipine administration significantly improved blood flow in ischemic state (Tachibana, 1990; Mitchell et al., 1992).

Nitrate drugs, such as nitroglycerin and isosorbide dinitrate, stimulate guanylate cyclase in vascular smooth muscle cells *via* nitrogen oxide. At low doses, nitrates dilate the vessels in the venous system and at high doses dilate the arterial system vessels to reduce resistance. They provide stress relief (by pulmonary capillary pressure reduction) and afterload relief (by mild elevation of cardiac output and decrease in peripheral vascular resistance). Moreover, nitrates are widely used in heart failure caused by ischemic heart disease for their coronary artery dilation effect.

Dihydropyridine (DHP) calcium antagonists, such as nicardipine, relax vascular smooth muscles by blocking the membrane voltage-dependent L-type calcium channels involved in the influx of extracellular calcium ions. They are used to treat hypertension by relaxing muscles and reducing peripheral vascular resistance. Their main pharmacological actions include coronary and peripheral vasodilator activity, cardiac contractility, and suppression of impulse conduction system. However, cardiac suppression is hardly seen at clinical doses. Nicardipine has excellent organ blood flow maintenance effect and is used in cases of organ failure due to hypertension. Among DHP calcium antagonists, nicardipine has a short onset time and half-life. It is used for hypertensive emergencies and is highly specific for cerebral blood vessels (Takenaka and Handa, 1979).

One common pharmacological effect of the three selected agents (isosorbide dinitrate, nitroglycerine, and nicardipine) is dilation of the coronary artery, which we suggest contributed to the improvement in survival rate after cardiac arrest in this study. Treatment of patients with cardiac arrest involves management of blood pressure; vasoconstrictors such as adrenaline and noradrenaline are commonly administered for this purpose. However, no sufficient evidence is available to support the effect of vasoconstrictors on the survival and discharge rate of adult patients after resumption of heart rate; excessive administration may lead to decreased blood flow. The prognosis improvement could have resulted from increased oxygen supply to the heart through the dilation of the coronary artery, relief in the heart afterload, and the increase in whole organ blood flow to the heart.

In addition, nicardipine has been shown to directly dilate cerebral blood vessels (Takenaka and Handa, 1979) and nitroglycerin has been reported to improve neurologic prognosis after cardiac arrest in animal studies (Chen et al., 2011). Irreversible damage to cranial nerves in post-cardiac arrest syndrome (PCAS), reported in most patients with cardiac arrest, is a very important risk factor for long-term poor prognosis of cardiac arrest patients. Thus, it can be suggested that increased cerebral blood flow, which suppresses irreversible damage to the cranial nerves associated with circulatory failure, also contributed to the improvement of long-term outcomes.

This study has several limitations. First, we could not evaluate the effect of drugs not approved in Japan. Because the JMDC claims database contains only Japanese claim data, we could only include drugs approved in Japan in the analysis. Thus, we may have missed promising drug candidates, which should be considered in future studies. Second, we defined each comorbidity based on the description in the claim data. However, the diagnostic methods and diagnosis criteria could be inconsistent, and, thus, the quality of diagnosis might be inconsistent. Third, the JMDC claims database does not provide some information, such as the cause of cardiac arrest, the quality of cardiopulmonary resuscitation, medication adherence, the dose and duration of drug administration, and clinical laboratory test results. In the studies using propensity scores, all factors affecting treatment allocation should be investigated and included as covariates. However, propensity score matching cannot be used as a substitute to the randomized comparison trials, which can balance out unknown covariates. In future, prospective observational studies should be conducted and information on background factors related to cardiac arrest should be collected.

## Conclusion

Three drugs (isosorbide dinitrate, nitroglycerine, and nicardipine) were identified in this study as candidate novel therapeutic agents to improve the prognosis of cardiac arrest patients. These results could prove to be valuable in therapeutic drug development for cardiac arrest patients because it is difficult to conduct clinical trials in these patients due to the urgency of their treatment. Moreover, the procedure used for the identification of candidate drugs in the present study is a very useful method that combines multifaceted evaluation using drug discovery tools and claims databases. To confirm the usefulness of the results and method used in this study and to use the three identified drugs for their therapeutic effect in cardiac arrest patients, it is necessary to validate these results in clinical settings.

## Data Availability Statement

All datasets generated for this study are included in the article/**Supplementary Material**.

## Ethics Statement

This study was conducted in keeping with the Ministry of Health, Labour, and Welfare’s Ethical Guidelines for Epidemiological Research. It was approved by the Okayama University Graduate School of Medicine, Dentistry, and Pharmaceutical Sciences and Okayama University Hospital Ethics Committee (No. 1706-022-001) and conformed to the tenets of the Declaration of Helsinki. Since this study was an observational study with anonymized information, with no treatment intervention and no collection of human samples, obtainment of informed consent was exempted.

## Author Contributions

YZ contributed to the conception and study design, data acquisition, statistical analysis, interpretation of the data, and the revision of the manuscript. TK and TN contributed to the interpretation of the data and the revision of the manuscript. YS, YI-I, MM, AO, KH, TI, YK, HH, NO, MG, KeT, MC, KoT, SH, MK, and KI contributed to the interpretation of the data and the critical review of the manuscript.

## Funding

This research was supported by the Japan Research Foundation for Clinical Pharmacology Grant (grant number 2018A10) and the Japan Society for the Promotion of Science (JSPS) KAKENHI (grant number 18K06785).

## Conflict of Interest

The authors declare that the research was conducted in the absence of any commercial or financial relationships that could be construed as a potential conflict of interest.
