# Supplementary material for: Search for Therapeutic Agents for Cardiac Arrest Using a Drug Discovery Tool and Large-Scale Medical Information Database
Source: Front Pharmacol. 2019 Nov 8;10:1257. doi: 10.3389/fphar.2019.01257 (PMC6857070; doi:10.3389/fphar.2019.01257)
Supplement: Supplementary file 1 [file DataSheet_1.docx]

***Supplementary Material***

**Supplementary Table 1. ATC code using extraction of candidate drug**

| ATC code | Classification |
| --- | --- |
| C01B | Cardiovascular system / heart disease drug / antiarrhythmic drug (class I and III) |
| C01DA | Cardiovascular system / cardiac disease treatment / cardiovascular vasodilator / organic nitrate |
| C02CA | Cardiovascular system / antihypertensive drug / anti-adrenergic drug (peripheral action type) / alpha adrenergic receptor agonist |
| C02CC | Cardiovascular system / antihypertensive / antiadrenergic drug (peripheral action type) / guanidine derivative |
| C04 | Circulatory system / peripheral vasodilator |
| C08 | Circulatory system / calcium channel blocker |

Abbreviation: ATC, Anatomical therapeutic chemical classification

**Supplementary Table 2. Patient information**

| Column name | Column explanation | Data form |
| --- | --- | --- |
| ID | A code uniquely assigned to each patient by JMDC | Alphanumeric |
| Birth date | Patient's date of birth | yyyymm |
| Gender | Patient gender (male, female) | A string |
| Family division | Patient's family division (person, family) | A string |
| Join date | Date of joining the health insurance | yyyymm |
| Withdrawal date | Year of withdrawal from the health insurance | yyyymm |
| Insurance withdrawal due to death | If the reason for withdrawal is death, code number 1 is assigned | Number |
| JMDC data conversion start date | Date when JMDC started to convert the health insurance data | yyyymm |
| JMDC data conversion end date | Date when JMDC ended to convert the health insurance data | yyyymm |

Abbreviation: JMDC, Japan medical data center

**Supplementary Table 3. Receipt information**

| Column name | Column explanation | Data form |
| --- | --- | --- |
| Receipt ID | Code uniquely assigned to each receipt by JMDC | Alphanumeric |
| Subscriber ID | A code uniquely assigned to each patient by JMDC | Number |
| Receipt type | Types of receipt (hospital, DPC, out of hospital, dispensing) | A string |
| Medical examination date | Date of medical examination | yyyymm |
| Medical facility ID | A code uniquely assigned to a medical facility by JMDC | Number |
| Medical department major Classification | The medical department described in the receipt is classified in the JMDC medical department master major classification | A string |
| Medical department middle Classification | The medical department described in the receipt is classified in the JMDC medical department master middle class classification | A string |
| Medical practice days | Days of medical practice | Number |
| Total medical fee point | Medical expenses per receipt (unit: points) | Number |

Abbreviation: JMDC, Japan Medical Data Center

**Supplement Table 4. Facility information**

| Column name | Column explanation | Data form |
| --- | --- | --- |
| Medical facility ID | A code uniquely assigned to a medical facility by JMDC | Number |
| Number of beds | Number of beds in the facility (0-19, 20-99, 100-199, 200-299, 300-499, 500+) | Alphanumeric |
| HPGP division | HP (more than 100 beds), GP (less than 99 beds) | A string |
| Medical department major classification | First medical treatment department of the facility is classified in the JMDC medical department master major classification | A string |
| Medical department middle classification | First medical treatment department of the facility is classified in the JMDC medical department master class classification | A string |
| Management body | A classification defined uniquely by JMDC from management body data of hospital information.  Public hospitals: more than 20 beds at national hospitals and prefectural and municipal hospitals  University hospitals: National public and private university hospitals with medical department (does not include dental department single department hospital)  Other hospitals: Public hospitals, hospitals with more than 20 beds that do not apply to university hospitals  Clinic: Clinic with over 19 beds | A string |
| Clinical training hospital | Clinical training hospital designated by the Ministry of Health, Labor and Welfare. Updated annually from the Ministry of Health, Labor and Welfare website.  1 flagship type, 2 cooperation type, 3 flagship and cooperation type | Number |
| Home care support clinic | A facility that reports on home care support clinics to the prefectural social insurance office. (1: applicable, 0: not applicable)  JMDC investigates disclosure requests to the Prefectural Social Insurance Office once a year. | Number |
| Regional medical support hospital | Facilities approved by the prefectural governor as a regional medical support hospital. (1: applicable, 0: not applicable)  Updated annually from the Ministry of Health, Labor and Welfare website. | Number |
| Cancer medical treatment cooperation base hospital | Regional and prefectural cancer treatment base hospitals designated by the Minister of Health, Labor and Welfare. (1: prefecture, 2: area)  Updated annually from the Ministry of Health, Labor and Welfare website. | Number |
| DPC introduced medical institutions | Facility that calculates medical expenses by DPC. (1: applicable, 0: not applicable) Updated annually from the Ministry of Health, Labor and Welfare website. | Number |
| Special function hospital | A specific functional hospital designated by the Ministry of Health, Labor and Welfare. (1: applicable, 0: not applicable)  Updated annually from the Ministry of Health, Labor and Welfare website. | Number |

Abbreviation: JMDC, Japan Medical Data Center; DPC, Diagnosis procedure combination

**Supplementary Table 5. Doctor information**

| Column name | Column explanation | Data form |
| --- | --- | --- |
| Doctor ID | Code uniquely assigned by JMDC, with medical facilities and doctors as keys | Number |
| Medical department major classification | The medical department of the doctor is classified in the medical department major classification | A string |
| Medical department middle classification | The medical department of the doctor is classified in the medical department middle class | A string |

Abbreviation: JMDC, Japan Medical Data Center

**Supplementary Table 6. Disease information**

| Column name | Column explanation | Data form |
| --- | --- | --- |
| Receipt ID | A code uniquely assigned to each receipt by JMDC | Alphanumeric |
| Subscriber ID | A code uniquely assigned to each patient by JMDC | Alphanumeric |
| Receipt type | Types of receipt (hospital, DPC, out of hospital, dispensing) | A string |
| Medical examination date | Date of medical treatment | yyyymm |
| Medical facility ID | A code uniquely assigned to a medical facility by JMDC | Number |
| ICD 10 major classification  code | Major classification code of international disease classification | Alphanumeric |
| ICD 10 major classification  name | Major classification name of international disease classification | A string |
| ICD 10 middle classification  code | Classification code of international disease classification | Alphanumeric |
| ICD 10 Middle Class Name | Intermediate classification name of international disease classification | A string |
| ICD10 minor classification code | International Classification of Diseases Classification Code | Alphanumeric |
| ICD 10 minor category name | Subclass name of international disease classification | A string |
| ICD 10 fine classification Code | International Classification of Diseases Classification Code | Alphanumeric |
| ICD 10 fine classification name | Subclassification name of international disease classification | A string |
| Standard disease name | Name of the disease created by Medical Information System Development Center (MEDIS- DC) | A string |
| Doubt flag | Flag when there is a statement of doubt in the illness (suspect: 1, confirmed: 0) | Number |
| Medical examination start date | Date when medical care for the disease was started at the medical facility | yyyymmdd |
| Outcome | 0. Continue 1. Cure 2. Death 3. Stop 4. Change doctor NULL. Unknown | Number |

Abbreviation: JMDC, Japan Medical Data Center; DPC, Diagnosis procedure combination; ICD-10, International Statistical Classification of Diseases and Related Health Problems-10

**Supplementary Table 7. Drug information**

| Column name | Column explanation | Data form |
| --- | --- | --- |
| Receipt ID | A code uniquely assigned to each receipt by JMDC | Alphanumeric |
| Item ID | Unique ID uniquely assigned to the record | Number |
| Subscriber ID | A code uniquely assigned to each patient by JMDC | Alphanumeric |
| Receipt type | Types of receipt (hospital, DPC, out of hospital, dispensing) | A string |
| Medical examination date | Date of medical treatment | yyyymm |
| Medical facility ID | A code uniquely assigned to a medical facility by JMDC | Number |
| Doctor ID | A code uniquely assigned by JMDC, with medical facilities and doctors as keys | Number |
| ATC major classification code | Major classification code of drug classification method prepared by EphMRA | Alphanumeric |
| ATC major name | Major classification name of drug classification method prepared by EphMRA | A string |
| ATC middle classification code | Middle classification code of drug classification method prepared by EphMRA | Alphanumeric |
| ATC middle class name | Middle class name of the drug classification method that EphMRA makes | A string |
| ATC minor classification code | Classification code of drug classification method prepared by EphMRA | Alphanumeric |
| ATC minor category name | Minor classification name of drug classification method prepared by EphMRA | A string |
| ATC “sub-classification code | Detailed classification code of drug classification method prepared by EphMRA | Alphanumeric |
| ATC Subclass Name | Subclassification name of the drug classification method that EphMRA makes | A string |
| Ingredient name | Name of the substance that constitutes the medicine | A string |
| brand name | Name of drug name excluding dosage form and potency information | A string |
| Drug name | Sales name of each pharmaceutical company | A string |
| Standard unit | Unit for drug price calculation | A string |
| Drug price reference listing Drug code | Classification code of Ministry of Health, Labor and Welfare | Alphanumeric |
| Generic flag | Flag for generic product (Original: 0, Generic: 1) | Number |
| Prescription date | DPC coding data and medical receipt date information, dispensing receipt dispensing date | Number |
| Daily dose per prescription | Daily dose of medicine prescribed for each medical treatment (solution, patch, single dose of ointment) | Number |
| Dosage unit | Unit when dose is calculated by JMDC definition | A string |
| Administration days per prescription | Administration days of medicine prescribed for each medical treatment (solution, patch, ointment is described by frequency of administration) | Number |
| Dosage | 1 dose per prescription × days of administration | Number |
| Formulation type major classification | Drug type major classification by Ministry of Health, Labor and Welfare | A string |
| Formulation type middle classification | Drug type middle classification by Ministry of Health, Labor and Welfare | A string |
| Simultaneous ID | Unique ID when the drug is co-prescribed within the same medical care category | Number |
| Medicines to be taken as needed flag | Flag of medicines to be taken as needed (regular use: 0, as needed: 1) | Number |
| Medical treatment division | Classification about administration situation of medicine described in medical receipt  14: at home, 20: medication（21: internal use, 22: as needed use, 23: external use）, 30: injection (31: subcutaneous/intramuscular injection, 32: intravenous injection, 33: other route)  40: treatment, 50: surgery, 54: anesthesia, 60: medical examination, 70: diagnostic imaging, 80: other | Number |
| Drug price | Price per dosage unit | Number |
| Dispensing fee | Dispensing receipt only | Number |
| Drug fee | Dispensing receipt only | Number |
| Additional fee | Dispensing receipt only | Number |

Abbreviations: JMDC, Japan Medical Data Center; DPC, Diagnosis procedure combination; ATC, Anatomical therapeutic chemical classification; EphMRA, European Pharmaceutical Market Research Association

**Supplementary Table 8. Medical practice information**

| Column name | Column explanation | Data form |
| --- | --- | --- |
| Receipt ID | A code uniquely assigned to each receipt by JMDC | Alphanumeric |
| Item ID | Unique ID assigned to the record | Number |
| Subscriber ID | A code uniquely assigned to each patient by JMDC | Alphanumeric |
| Receipt type | Types of receipt (hospital, DPC, out of hospital, dispensing) | A string |
| Medical examination date | Date of medical treatment | yyyymm |
| Implementation date | Date of implementation (DPC only) | yyyymmdd |
| Medical facility ID | A code uniquely assigned to a medical facility by JMDC | Number |
| Medical treatment major classification name | Major classification of medical treatment score chart | A string |
| Medical treatment minor classification name | Minor classification of medical treatment score chart | A string |
| Medical treatment fine classification name | Fine classification of medical treatment score chart | A string |
| Medical treatment score quick reference classification code | Code described in the medical score chart | Alphanumeric |
| Standardized medical practice ID | Code of medical treatment practice described in master of medical care | Number |
| Standardized medical practice name | Name of medical care described in master of medical care | A string |
| Number of times | Number of times the medical service was performed | Number |
| Simultaneous ID | Unique ID when it is carried out simultaneously in the same medical care division | Number |
| Scene of medical practice | Classification based on the scene of the medical practice described in medical receipt  11: first examination, 12: re-examination, 13: medical management, 14: at home, 20: medication (21 internal use, 22 as needed use, 23 external use, 24 dispense, 25 prescriptions, 26 narcotics, 27 basic charge of dispense), 30: injection (31 subcutaneous/intramuscular injection, 32 intravenous injection, 33 other route) 40: treatment, 50: surgery, 54: anesthesia, 60: medical examination, 70: diagnostic imaging, 80 other, 90: hospitalization, 92: specific hospitalization charge, 97: diet and daily care | Number |
| Medical fee point | Medical fee point of treatment | Number |

Abbreviation: JMDC, Japan Medical Data Center; DPC, Diagnosis procedure combination

**Supplementary Table 9. Covariates used for adjustment**

| Covariate | Extraction condition | Date condition |
| --- | --- | --- |
| Sex | Sex in patient information |  |
| Age | Difference between cardiac arrest day and birthday |  |
| **Medical history** | | |
| Ischemic heart disease | ICD-10 code "I20-I25" | Until the previous month of cardiac arrest |
| Cerebrovascular disease | ICD-10 code "I60-I69" | Until the previous month of cardiac arrest |
| Kidney disease | ICD-10 code "N00-N08" or "N10-N16" or "N17-N19" | Until the previous month of cardiac arrest |
| Liver disease | ICD-10 code "K70-K77" | Until the previous month of cardiac arrest |
| Chronic lung disease | ICD-10 code "J40-J47" | Until the previous month of cardiac arrest |
| Heart failure | ICD-10 code "I50" | Until the previous month of cardiac arrest |
| Diabetes mellitus | ICD-10 code "E10-E14" | Until the previous month of cardiac arrest |
| High blood pressure | ICD-10 code "I10-I15" | Until the previous month of cardiac arrest |
| Hyperlipidemia | ICD-10 code "E785" | Until the previous month of cardiac arrest |
| Malignant neoplasm | ICD-10 code "C00-C14" or "C15-C26" or "C30-C39" or "C40-C41" or "C43-C44" or "C45-C49" or "C50-" or "C51-C58" or "C60-C63"  or "C64-C68" or "C69-C72" or "C73-C75" or "C76-C80" or "C81-C96" or "C97-" or "D00-D09" | Until the previous month of cardiac arrest |

| **Emergency treatment factor** | | |
| --- | --- | --- |
| Out-of-hospital cardiac arrest | Medical treatment chart quick index classification code "A300" | Same day as cardiac arrest |
| Average number of defibrillations | Medical treatment chart quick index classification code "J047" | Same day as cardiac arrest |
| Tracheal intubation | Standardized practice name "tracheal intubation for life saving" | Same day as cardiac arrest |
| Artificial respiration | Medical treatment chart quick index classification code "J045" | Same day as cardiac arrest |
| Hypothermia | Medical treatment chart quick index classification code "L008-2" | Same day as cardiac arrest |
| Adrenaline | Ingredient name "adrenaline" and, major classification "injectable" | Same day as cardiac arrest |
| Vasopressin | Ingredient name "vasopressin" | Same day as cardiac arrest |
| Amiodarone | Ingredient name "amiodarone" and dosage form "injectable" | Same day as cardiac arrest |
| Lidocaine | Name of drug "xylocaine 2% for intravenous injection" or "lidocaine 2% syringe for intravenous injection "Terumo"" | Same day as cardiac arrest |
| Nifekalant | Ingredient name "nifekalant" | Same day as cardiac arrest |

Abbreviation: ICD-10, International Statistical Classification of Diseases and Related Health Problems-10
